# Patient satisfaction with specialized mental health service for obsessive-compulsive disorder

**DOI:** 10.1186/1744-859X-12-41

**Published:** 2013-12-16

**Authors:** Paraskevi Mavrogiorgou, Frauke Siebers, Georg Juckel, Thorsten Kienast

**Affiliations:** 1 Department of Psychiatry, Ruhr University Bochum, LWL-Universitätsklinikum, Alexandrinenstr 1, Bochum 44791, Germany; 2 Department of Psychiatry and Psychotherapy, Charite Campus Mitte, Universitätsmedizin Berlin, Chariteplatz 1, Berlin 10117, Germany

**Keywords:** Satisfaction, Verona service satisfaction scale, OCD

## Abstract

**Background:**

Patient satisfaction is an important outcome variable that is increasingly used in mental health service evaluation. There are no results available for patients with obsessive-compulsive disorder (OCD) yet.

**Methods:**

Using the Verona Service Satisfaction Scale, patient satisfaction with a specialized mental health service was examined in patients with OCD.

**Results:**

OCD patients were overall satisfied with the professional help provided, whereas satisfaction with the professional involvement of relatives within the treatment and health care process was found to be quite low. Patients with more severe OCD, as measured by the Yale-Brown Obsessive-Compulsive Scale, as well as chronically ill and more disabled patients were more likely to be dissatisfied with the overall care they received. Patient satisfaction plays an important role in the long-term course of an illness such OCD. This seems to be decreased so longer illness is not or badly treated. There is a stronger need for involvement of family members in the treatment and health care of patients with OCD.

**Conclusions:**

More OCD-specific treatment offers have to be established for patients with this long-term illness such as psychotherapy in groups.

## Introduction

Obsessive-compulsive disorder (OCD) is a common psychiatric disorder which affects 1%–3% of the population [1]. Patients with OCD suffer from recurrent, unwanted thoughts (obsessions) and repetitive, ritualized behaviour (compulsions), which are often intended to neutralize anxiety induced by the obsessions. OCD often evolves as a chronic illness [2] with severe occupational and social impairment. In the last two decades, various efficacious pharmacological and psychotherapeutic treatments for OCD have been extensively studied and well established [3]. A combination of cognitive and behavioural therapy [4,5] and pharmacological agents, preferentially serotonin reuptake inhibitors [6,7], improves OCD symptoms in about 70% of patients [8]. However, up to 40%–60% of OCD patients do not have a satisfactory response after adequate treatment [9]. This high rate of non-responders and current neurobiological findings offers that OCD is a pathogenetic and phenotypic highly heterogeneous disorder and possibly composed of many different subtypes [10,11].

Furthermore, treatment non-response could be caused by many factors, but most specifically by different or difficult modalities and conditions of treatment, e.g. methodological non-adherent treatment. Although tremendous progress in the treatment of OCD was achieved by research, help-seeking behaviour of the patients with OCD as well as the daily therapeutic practice seems to remain almost unchanged. Only few studies report that the majority of patients with OCD attend first professional treatment after 6–10 years from onset of OCD symptoms [12-14]. In addition, Külz et al. [15] found in a survey of 177 psychotherapists that indicated specific treatment of OCD patients was conducted in only very few cases. Furthermore, most of these therapists (75%) had given treatment in only three patients per year.

Reasons for this delayed help-seeking behaviour in OCD patients which results in a bad outcome and course of illness are rarely investigated yet. The first evidence could be accomplished by a study of 23 patients with OCD by using a problem-orientated interview [13]. Not only the failing knowledge and missing experience with evaluated OCD therapies on the side of the professionals, but also trivialisation of complaints on the side of the patients and their relatives, could be identified as major constraints for early recognition and intervention. In a broader view, limited knowledge about any illness, its treatment possibilities and specific help offered by the health care system are great barriers for taking and receiving professional support in general [16,17]. Furthermore, shame, awkwardness and anxiety concerning stigmatization are also obstacles preventing patients to enter effective treatment [18]. There is a large need for research to identify further barriers for treatment in patients with OCD, which must be overwhelmed in order to provide sufficient help for the patients.

A core parameter for the positive evaluation of a mental health care system, i.e. psychiatric outpatient clinic and its treatment options, is patient satisfaction. However, various study results with regard to patient satisfaction within psychiatric services and treatments are ambiguous and not comparable. Their major limitations are methodological such as heterogeneous definition of patient satisfaction and usage of different non-standardized assessment tools and scales. Notably, the working group around Ruggeri has developed the meanwhile well-validated and in several European languages translated standardized Verona Service Satisfaction Scale (VSSS). This scale measures patient satisfaction; however, it is not only limited within specific psychiatric in- and outpatient care but also with respect to required complementary health care domains as those of caregivers, relatives and professionals besides the estimation of the patient itself [19-21]. Whereas to our knowledge no study has yet investigated a useful application of this scale in patients suffering from OCD, there are several precursor studies in patients with schizophrenia [19,22-24] pinpointing to the assumption that this scale may give an appropriate standard tool to investigate patients' satisfaction even for the population of patients with OCD.

Investigations of patient satisfaction in OCD are still rarely a study assessing patients' satisfaction with the VSSS which was not conducted yet in patients with OCD to our knowledge. The aim of this study was therefore to examine the satisfaction of patients with OCD by using the VSSS during a treatment in a specialized mental health outpatient service for OCD and to determine this in regard to the current psychopathology and socio-demographic parameters.

## Methods

### Study sample

Forty patients with unequivocal diagnosis of obsessive-compulsive disorder were recruited from the outpatient clinic for OCD at the Department of Psychiatry, Ruhr University Bochum. The diagnosis was based on the diagnostic criteria of the Diagnostic and Statistical Manual of Mental Disorders, fourth edition (DSM-IV) and ICD10.

Exclusion criteria were organic psychiatric disorders or recent concomitant neurological or other medical disorders and the presence of severe alcohol or substance abuse. No patient met the criteria for Tourette syndrome. Comorbid major depression and anxiety disorders were not considered as exclusion criteria. Table 1 shows the socio-demographic and clinical data of the 40 patients included in the study. Most patients received a variety of constant medications including antidepressant and/or an adjunct antipsychotic agent during observation period (Table 2). Cognitive behavioural therapy was not considered as an exclusion criterion.

**Table 1:** Socio-demographic and clinical characteristics of OCD patients

	**OCD** (***n*** = **40**)
Gender (*n* (%))	
Female	23 (57.5)
Male	17 (42.5)
Age (mean (SD), range, years)	39.4 (10.1), 22–55
Marital status (*n* (%))	
Married/cohabitating	25 (62.5)
Single	15 (37.5)
Education (*n* (%))	
Upper grade	26 (65)
Middle grade	7 (17.5)
Lower grade	7 (17.5)
Occupational status (*n* (%))	
Employed	19 (47.5)
Student	6 (15)
Homemaker	2 (5)
Unemployed	3 (7.5)
Retired, unable to work (sickness)	10 (25)
Duration of illness (mean (SD) years)	17.5 (11)
Age of onset (mean (SD), years)	21.9 (10.3)
HAMD score (mean (SD))	13.43 (7.9)
BDI (mean (SD))	15.08 (11.1)
Y-BOCS—Obsessions (mean (SD))	9.8 (5.1)
Y-BOCS—Compulsions (mean (SD))	9.2 (4.9)
Y-BOCS total score (mean (SD))	19 (9.3)
MOCI (mean (SD))	13.6 (4.9)
STAI I (mean (SD))	44.5 (12.3)
STAI II (mean (SD))	50.3 (11.8)
CGI (mean (SD))	4.5 (1.0)
PSP (mean (SD))	45 (18.5)

*HAMD* Hamilton Depression Scale, *BDI* Beck Depression Inventory, *Y-BOCS* Yale-Brown Obsessive-Compulsive Scale, *MOCI* Maudsley Obsession-Compulsive Inventory, *CGI* Clinical Global Impressions, *STAI* State-Trait Anxiety Inventory, *PSP* Personal and Social Performance Scale, *SD* standard deviation.

**Table 2:** Psychopharmacological medication and daily dose

	**Number** (%)	**Daily dose** (**mg**)
AD**-**monotherapy	24 (60)	
Citalopram	5 (12.5)	28 (20–40)^a^
Fluoxetine	6 (15)	21.7 (10–40)^a^
Paroxetine	5 (12.5)	23 (15–80)^a^
Sertraline	4 (10)	212.5 (150–300)^a^
Clomipramine	1 (2.5)	25
Duloxetine	1 (2.5)	60
Bupropion	2 (5)	150
Combination therapy	12 (30)	
Clomipramine + quetiapine	1 (2.5)	175 + 200
Clomipramine + aripiprazole	1 (2.5)	150 + 50
Citaloprame + promethazine	1 (2.5)	40 + 100
Citaloprame + buspirone	1 (2.5)	20 + 5
Citaloprame + aripiprazole	1 (2.5)	80 + 10
Fluoxetine + quetiapine	1 (2.5)	40 + 100
Sertraline + quetiapine	2 (5)	125 + 62.5
Sertraline + aripiprazole	1	200 + 7.5
Trazodone + quetiapine	1 (2.5)	300 + 200
Trimipramine + lithium	1 (2.5)	250 + 1.125
Venlafaxine + promethazine	1 (2.5)	150 + 50
None	4 (10)	

^a^Values are presented as median (range) unless otherwise specified.

### Measures

The severity of obsessive-compulsive symptoms was assessed by the Yale-Brown Obsessive-Compulsive Scale (Y-BOCS) [25,26] and Maudsley Obsessive-Compulsive Inventory (MOCI) [27]. To validate the presence of OCD (sub)symptoms, we use the Yale-Brown Obsessive-Compulsive symptom checklist.

The severity of depressive symptoms was assessed using the Hamilton Depression Rating Scale (HAMD) [28] and self-ratings with Beck's Depression Inventory (BDI) [29]. Anxiety symptoms were measured using the State-Trait Anxiety Inventory (STAI I and II) [30,31].

The overall severity of the psychiatric disorder was quantified using the Clinical Global Impression (CGI) score [32].

Patients' psychosocial functioning was measured by the Personal and Social Performance Scale (PSP) [33].

Patient satisfaction was assessed using the German Version of the Verona Service Satisfaction Scale (VSSS-54), an instrument developed by careful translation and cultural adaptation from the original VSSS [21]. It is designed for use in comparative cross-national research projects as well as in routine clinical practice in mental health services across Europe and has been shown to have a good validity and reliability [20]. VSSS-54 consists of 54 items, which conceptually covers seven dimensions: (1) overall satisfaction, (2) professional skills and behaviour, (3) information, (4) access, (5) efficacy, (6) type of intervention and (7) relative's involvement. For items 1–40, satisfaction ratings are on a 5-point Likert scale (terrible < mostly unsatisfactory < mixed < mostly unsatisfactory < excellent). Items 41–54 consist of three questions: First, the subject is asked if he/she has received the specific intervention (question A). If the answer is ‘yes’ , he/she is asked his/her satisfaction on a 5-point Likert scale (question B). If the answer is ‘no’ , the subject is asked question C: ‘Do you think you would have liked to receive intervention x?’ (6 = no, 7 = do not know, 8 = yes). These questions permit the estimation of the subjective degree of satisfaction both with the interventions provided and with the professional's decision not to provide an intervention. The latter may be considered a measure of underprovision of care, from the patient's point of view [20]. Missing values were set as ‘0’ in calculating the seven VSSS dimensions as well as the total score. The VSSS-54 is simply designed and can be completed in 30 min without prior training.

Psychopathological interviews were performed by an experienced psychiatrist (PM), and separate interviews for the socio-demographic variables as well as for VSSS-54 were conducted by an independent second rater (FS). Both were conducted in the outpatient clinic.

### Statistical analysis

Descriptive statistics are given as mean values, standard deviation and range. Statistical analyses were performed by appropriate parametric or nonparametric tests (*t* test, ANOVA and Pearson or Spearman correlation coefficients) with SPSS 21.0 for Windows. Statistical significance was *p* < 0.05. A value of *p* < 0.10 was regarded as statistical tendency.

## Results

### Clinical features

The socio-demographic and clinical characteristics for the 40 patients with OCD are summarized in Table 1. The mean age at the time of interview was 39.4 years (range 22–55 years) and the mean age at onset of OCD symptom was 21.9 years (range 6–42 years). Of the 40 OCD patients, 18 (45%) had an early onset, with a symptom manifestation before their 18th birthday. The mean duration of illness was 17.5 years (range 2–43 years). At the time of interview, 25 (62.5%) of the patients were married or cohabitating, and the remaining 15 (37.5%) were single. The sample was well educated, 33 (82.5%) had a high or middle educational degree. At the time of the interview, 67.5% (*n* = 27) of the sample was employed, while 32.5% reported being unable to work mostly due to the psychiatric illness.

Most patients were already undergoing pharmacological and/or psychological treatment. For example, 24 (60%) of the patients were being medicated with an antidepressant agent, mostly serotonin reuptake inhibitors (SSRI) as a monotherapy, and 12 (30%) of the patients were being medicated with a combination of an antidepressant and an antipsychotic agent (Table 2). The mean total Y-BOCS score was 19.0 ± 9.3 indicating moderate OCD. The severity of the depressive symptoms according to HAMD-21 was mild with a total mean score of 13.4 ± 7.9. The mean total PSP score was 45.0 ± 18.5, indicating marked difficulties in two or more of areas of psychosocial functioning.

### Service satisfaction

The responses to the seven dimensions and 54 items of the VSSS-54 are presented in Table 3 as mean (SD) values for the OCD patients studied. The higher the score, the more satisfied the patients were. The mean VSSS-54 total score was 3.2 (SD = 0.5) indicating a mixed satisfaction. The highest mean scores were found in ‘overall satisfaction’ (4.1 ± 0.7) and ‘information’ (3.8 ± 0.8). We found in the dimension ‘relative's involvement’ the lowest mean score of 1.4 (SD = 1.9) indicating a very weak satisfaction. When looking at individual items of this dimension (Table 4), most of them exhibited middle until high values. This implies that those OCD patients whose relatives were involved within the therapeutic process were highly satisfied. The low total score of the dimension relative's involvement revealed that many of the OCD patients have not chosen the involvement of their relatives, although they found it necessary and desirable. Moreover, the lowest mean scores of individual items were found in ‘group sessions’ (3.3 ± 1) and ‘helping to establish good relationships outside family’ (3.4 ± 1.1).

**Table 3:** **Values of VSSS-54 total score and the seven dimensions in OCD patients (*n* = 40)**

	**Mean (SD)**	**Range**
Overall satisfaction	4.1 (0.7)	2.7–5.0
Professionals skills	3.0 (0.8)	1.6–4.6
Information	3.8 (0.8)	1.3–5.0
Access	3.7 (0.6)	2.0–5.0
Efficacy	3.1 (0.8)	1.0–5.0
Types of intervention	3.2 (0.3)	2.4–3.8
Relative's involvement	1.4 (1.9)	0–5.0
Verona total score	3.2 (0.5)	2.2–4.2

*SD* standard deviation (1 = terrible; 5 = excellent).

**Table 4:** VSSS-54 items in OCD patients

**Dimension**	**Mean (SD)**
1. Overall satisfaction (3 items)	
Item 11: amount of help received	4.3 (0.7)
Item 20: kind of services	4.2 (0.8)
Item 21 service general sense	4.4 (0.5)
2. Professional's skills and behaviour (16 items)	
Item 2: behavior and manners of reception staff	3.7 (1.0)
Item 3: professionalism of psychiatrists/psychologists	4.8 (0.4)
Item 5: ability of psychiatrists/psychologists to listen	4.8 (0.4)
Item 6: personal manner of psychiatrists/psychologists	4.8 (0.7)
Item 7: punctuality of the professionals	4.2 (1.0)
Item 10: confidentiality and respect for patients rights	4.6 (0.5)
Item 16: thoroughness of psychiatrists/psychologists	4.7 (0.5)
Item 17: referring to general practitioner or other specialists	4.5 (0.5)
Item 18: cooperation between service providers	4.3 (0.7)
Item 22: professional competence of nurses/social workers	4.1 (0.8)
Item 25: personal manner of nurses/social workers	4.1 (0.7)
Item 28: nurses knowledge of patients medical history	3.6 (0.8)
Item 33: instruction on what to do between visits	3.9 (0.6)
Item 35: thoroughness of nurses/social workers	4.0 (0.6)
Item 37: ability of nurses/social workers to listen	3.9 (0.9)
Item 40: continuity of care	4.4 (0.9)
3. Information (3 items)	
Item 12: explanation procedures and approaches used	4.6 (0.6)
Item 19: publicity on mental health services offered	3.9 (0.9)
Item 29: information on diagnosis and prognosis	4.4 (0.6)
4. Access (2 items)	
Item 4: appearance, comfort level and physical layout	3.7 (0.7)
Item 8: costs of the service	3.9 (0.7)
5. Efficacy (8 items)	
Item 1: helping patient deal with problems	4.4 (0.6)
Item 9: attaining well-being and preventing relapses	4.2 (0.6)
Item 13: helping to relieve symptoms	4.1 (0.8)
Item 24: helping patient improve knowledge of his problems	4.4 (0.6)
Item 26: improving relationship between patient and relative	3.9 (0.9)
Item 31: helping to establish good relationships outside family	3.4 (1.1)
Item 34: helping to improve capacity to look after himself	3.6 (0.9)
Item 38: helping patient improve abilities to work	3.8 (0.8)
6. Type of intervention (17 items)	
Item 14: response to crisis during office hours	3.5 (1.0)
Item 15: response to emergencies during nights, weekends	5.0 (0)
Item 39: help for discomfort of side effects from medications	4.1 (0.7)
Item 41: medication prescription	4.0 (0.9)
Item 42: individual rehabilitation	3.9 (0.7)
Item 43: individual sessions	4.6 (0.8)
Item 44: compulsory treatment in hospital	4.0 (0.2)
Item 45: family sessions	3.8 (0.9)
Item 46: living in sheltered accommodation	3.9 (0.4)
Item 47: recreational activities in the service	3.7 (0.8)
Item 48: group sessions	3.3 (1.0)
Item 49: shelter work	3.7 (0.7)
Item 50: informal admission to hospital	4.0 (0.6)
Item 51: practical help by the service at home	3.8 (0.7)
Item 52: helping in obtaining welfare benefits	3.9 (0.7)
Item 53: help to find open employment	3.5 (0.9)
Item 54: recreational activities outside the service	3.8 (0.7)
7. Relative's involvement (5 items)	
Item 23: recommendations about how relative could help	4.3 (0.7)
Item 27: helping relative to deal better with patient's problems	4.0 (0.7)
Item 30: ability of psychiatrists/psychologists to listen to relative	4.4 (0.6)
Item 32: information to relative about diagnosis and prognosis	4.1 (0.9)
Item 36: helping relative improve understanding of patients problems	4.0 (0.9)

*SD* standard deviation (1 = terrible; 5 = excellent).

As shown in Table 5, the greater OCD symptom severity (Y-BOCS total score) was associated with lower mean score in the dimension ‘type of intervention’ (*r* = −0.430, *p* < 0.006), i.e. patients who were more ill were found to be less satisfied with this dimension. Especially, the highest significant negative correlation was found between the mean score of type of intervention and the Y-BOCS subscore for compulsion (*r* = −0.498, *p* < 0.001). Concerning the relation between psychopathology and service satisfaction, significant negative correlation between the depression scales (HAMD-21 and BDI) and the mean total score of type of intervention was found (*r* = −0.425, *p* < 0.006; *r* = −0.387, *p* < 0.014).

**Table 5:** Correlation coefficients between psychometric variables and VSSS-54

	**VS-T**	**OS**	**PR sk**	**IN**	**AC**	**EF**	**TI**	**RI**
YB-T	0.57	−0.038	0.129	0.027	−0.145	−0.152	−0.43**	0.269
YB-C	0.057	−0.050	0.070	0.011	−0.150	−0.083	−0.347*	0.0265
YB-O	0.045	−0.033	0.158	0.052	−0.156	−0.157	−0.498**	0.233
MOCI	0.119	0.064	0.143	0.045	−0.047	−0.114	−0.202	0.239
HAMD	0.079	0.038	0.219	−0.062	−0.121	0.012	−0.425**	0.244
BDI	−0.128	0.047	−0.017	−0.090	−0.208	−0.248	−0.387*	0.094
ST-I	0.134	−0.042	0.119	−0.036	0.008	−0.061	−0.249	0.317*
ST-II	−0.029	−0.090	0.215	−0.111	−0.179	−0.199	−0.210	0.188
CGI	0.010	−0061	0.066	−0.044	−0.220	−0.250	−0.407**	0.293
PSP	−0.001	0.140	−0.243	−0.059	0.006	0.046	0.305	−0.104

*p < 0.05; **p = 0.01.

*YB-T* Yale-Brown Obsessive-Compulsive Scale (total score), *YB-C* Yale-Brown Obsessive-Compulsive Scale (subcore compulsion), *YB-O* Yale-Brown Obsessive-Compulsive Scale (subcore obsessions), *MOCI* Maudsley Obsession-Compulsive Inventory, *HAMD* Hamilton Depression Scale, *BDI* Beck Depression Inventory, *CGI* Clinical Global Impressions, *STI/II* State-Trait Anxiety Inventory, *PSP* Personal and Social Performance Scale, *VS-T* Verona total score, *OS* overall satisfaction, *PR sk* professionals skills and behaviour, *IN* information, *AC* access, *EF* efficacy, *TI* type of intervention, *RI* relative's involvement.

There was no significant influence of co-variables such as age, suicide rate, psychotherapy parental occupational status and parental alcohol abuse on the VSSS-54. As shown in Table 6, male OCD patients had the tendency to be more satisfied in the type of intervention than female patients (*t* test, *p* = 0.065). OCD patients with non-academic occupational status were significantly more satisfied with the relative's involvement dimension compared to OCD patients with academic status (*p* = 0.019). In addition, patients with positive family history were significantly more satisfied than OCD patients without family history as indicated by the total VSSS-54 score (*p* = 0.048) as well as by the dimensions ‘professionals skills and behaviour’ (*p* = 0.076) and ‘types of intervention’ (*p* = 0.046). Furthermore, married OCD patients were significantly more satisfied with the type of intervention than OCD patients which live alone without a spouse or partner (*p* = 0.041). Concerning this dimension, more patients who were not characterized by a true OCD alone were more satisfied (*p* = 0.032). OCD patients who regularly consumed ethanol showed less satisfaction with the caring service (VSSS-54 total score) than the abstinent patients (*p* = 0.082).

**Table 6:** Influence of co-variables on VSSS-54

	**VS**-**T**	**OS**	**PR sk**	**IN**	**AC**	**EF**	**TI**	**RI**
Gender	n.s.	n.s.	n.s.	n.s.	n.s.	n.s.	*p* = 0.065	n.s.
Education	n.s.	n.s.	*p* = 0.076	n.s.	n.s.	n.s.	*p* = 0.068	*p* = 0.019
Occupation status	n.s.	n.s.	n.s.	*p* = 0.048	n.s.	n.s.	n.s.	*p* = 0.067
Marital status	n.s.	n.s.	n.s.	n.s.	n.s.	n.s.	*p* = 0.041	n.s.
Family history	*p* = 0.048	n.s.	*p* = 0.076	n.s.	n.s.	n.s.	*p* = 0.046	n.s.
Alcohol consumption	*p* = 0.082	n.s.	*p* = 0.004	*p* = 0.011	n.s.	n.s.	n.s.	n.s.
Comorbidity	n.s.	n.s.	n.s.	n.s.	n.s.	n.s.	*p* = 0.032	n.s.

*p* values are obtained using *t* test.

*n.s.* not significant, *VS-T* Verona total score, *OS* overall satisfaction, *PR sk* professionals skills and behavior, *IN* information, *AC* access, *EF* efficacy, *TI* type of intervention, *RI* relative's involvement.

There was a significant positive correlation between age of onset and total score on the VSSS-54 (*r* = 0.424, *p* = 0.006) and with the dimension professional skills and behaviour (*r* = 0.412, *p* = 0.008), although there was a significant negative correlation between duration of illness and VSSS total score (*r* = −0.443, *p* = 0.005) and the dimension professional skills and behaviour (*r* = −0.383, *p* = 0.016) and relative's involvement (*r* = −0.330, *p* = 0.040). Finally, the descriptive analysis of free answers provided by the patients to the last section of the VSSS, in which they were asked to state ‘The thing I liked the most…’ and ‘The thing I disliked the most…’ , revealed that competence concerning OCD in all aspects by the mental health service is very important positive aspect. In contrast, the failed offer of group therapy sessions focused on OCD as well as the not-sufficient involvement of relatives and care givers within the treatment and psychotherapy were often mentioned as less satisfactory.

## Discussion and conclusions

To our knowledge, this is the first study to examine the satisfaction of OCD patients by using the VSSS-54 during treatment in a specialized mental health outpatient service for OCD and to determine this in regard to the current psychopathological state as well as to socio-demographic parameters. Overall, outpatients with OCD were mixed satisfied with the help and support provided. In contrast, satisfaction with relative's involvement was low. Our findings are in accordance with the findings among patients with schizophrenia [19] and patients with depressive and bipolar disorder [34]. Specifically, the relative's involvement in the process of care and psychoeducation interventions is the satisfaction dimension in which mental health services in most domain regions show the worst performance [19].

On the other hand, OCD influences not only patients but also family members affected by the patients' behaviour; thus, several studies have investigated the impairment of family functioning as a potential risk factor as well as a moderator or treatment outcome measure in OCD [35,36]. For this reason, therapy plans should also include strained family interventions and thus may improve patients' (and families') satisfaction. Additional research concerning the content, process and effects of family interventions in patients with OCD is strongly warranted.

Our study has found that higher illness severity, as measured by Y-BOCS, is associated with lower professional medical service satisfaction, especially in the dimension type of interventions. Similar results of satisfaction concerning psychopathology have been found in previous studies in patients with schizophrenia [19,37]. Furthermore, in a study of patients with any psychiatric diagnosis, it has been found that patients with higher levels of psychopathology displayed lower satisfaction with the type of intervention received, independent from the psychiatric diagnosis respectively [38]. The previous literature concerning the effects of patient socio-demographic characteristics on service satisfaction remains inconclusive. On the one hand, no clear relationship has been found between satisfaction and psychosocial factors, such as marital status, occupation and education [39]; on the other hand, previous findings indicate that socio-demographic variables have only a modest impact in the various domains of satisfaction [19,34,38,40,41]. In line with these findings in the present study, we found that OCD patients without partner or spouse were more dissatisfied in the type of interventions, similar to the patients without positive family history. In contrast to the previous finding from Ruggeri et al. [38], we do not confirm that more socially disabled patients express lower satisfaction. In our study, OCD patients with higher educational and academic occupational status expressed lower satisfaction, especially with relative's involvement. This result presumably reflects the differences concerning the expectations and demands to the health care system between higher- and lower-educated patients with OCD. In general, there is a little agreement about the psychosocial factors that influence service satisfaction, and longitudinal studies will have to be carried out to investigate the role of these factors in determining the satisfaction with mental health service in the future.

In contrast to previous work that younger patients are less satisfied with mental health care [19,34,42,43], our data show that the actual age of the patients with OCD did not influence the results of VSSS-54. However, in the present study, a significant association has been found between the age of onset and satisfaction, with more dissatisfaction in the dimensions of professional skills and behaviour and relative's involvement in OCD patients with early onset. In this regard, the finding has to be discussed that patients with longer illness duration were in general less satisfied with the health care service, as reflected by a low total score of VSSS-54. They were also characterized by the lower rating of the dimension professional skills and behaviour as those with a shorter illness duration. This finding corresponds with several previous reports showing that patients with a long course of illness, i.e. long contact to the health care system, were often increasingly dissatisfied with the care and help they have received [38,44,45]. It can be speculated whether or not more chronic patients may have different expectations and needs as well as different help and support options as acutely ill patients. The cited studies provide hints that support of chronic patients with more intensive socio-therapeutic interventions and procedures could enhance satisfaction with the health care system.

In summary, it can be concluded that long-term ill and more disabled OCD patients are more likely to be dissatisfied with the care they received according to the findings in the VSSS. Since satisfaction with health care is an essential factor for therapeutic outcome and predictor of long-term prognosis of an illness, the results of the presented study underlie the need of early recognition and early intervention in patients with beginning OCD: Specialized centres for patients with OCD may offer a more systematic treatment with focus on OCD-specific type of interventions and involvement of relatives and care givers.

Limitations of this study include a number of methodological deficiencies. Firstly, our sample of OCD patients was not large enough to assess the relationships between VSSS-54 subdimensions and clinical subtypes of OCD (patients with only obsessions versus patients with only compulsions). Secondly, this study was performed in an intensive university outpatient setting with a higher personal resource, so the results may not be representative for standard outpatient clinic or psychiatrists and psychotherapists in practice, where most likely less severely impaired patients are treated. Additionally, our study is limited by the lack of a comparison group or matched healthy controls. Although the VSSS-54 is the currently best investigated and validated study for the assessment of patient satisfaction, the interviews in our specialized outpatient clinic for OCD with a wide regional catchment area using the VSSS were in parts difficult: Several patients did not participate at all treatment and health care possibilities as listed by the VSSS; therefore, they had no chance to judge these adequately. On the other side, there is an unclear interaction of the satisfaction and real participation in the offered services of the health care system [21]. It seems to be necessary to modify the VSSS according to the real treatment and health care service supplies in a definitive geographical region for patients with a definitive illness, in which some recent studies have begun to realize [41,43].

More OCD-specific treatment offers have to be established for patients with this long-term illness such as psychotherapy in groups. Further studies are needed to have a closer look on the correlational questions of what impact do the clinical changes have on the psychopathological symptoms and psychosocial functioning and on service satisfaction.

## Competing interests

The authors declare that they have no competing interests.

## Authors' contributions

PM and GJ carried out the design of the study. PM and FS performed the study by interviewing the patients. GJ, TK and PM performed the statistical analysis. TK conceived of the study and participated in its design and coordination. PM and GJ wrote the first draft. All authors read and approved the final manuscript.

## Declarations

We confirm all patient/personal identifiers have been removed or disguised so the patient/persons described are not identifiable and cannot be identified through the details of the study.
